# Auricular acupuncture with seed or pellet attachments for primary insomnia: a systematic review and meta-analysis

**DOI:** 10.1186/s12906-015-0606-7

**Published:** 2015-04-02

**Authors:** Ying Lan, Xi Wu, Hui-Juan Tan, Nan Wu, Jing-Jing Xing, Fu-Sheng Wu, Lei-Xiao Zhang, Fan-Rong Liang

**Affiliations:** Acupuncture and Tuina School/Third Teaching Hospital, Chengdu University of Traditional Chinese Medicine, No.37, Shierqiao Road, Jin Niu District, Chengdu, Sichuan 610075 China; VIP Ward of Acupuncture, First Teaching Hospital of Tianjin University of Traditional Chinese Medicine, No.314, An Shanxi Road, Nankai District, Tianjin, 300073 China; Sichuan 2nd Hospital of Traditional Chinese Medicine, No.20, Sidao Street, Qing Yang District, Chengdu, Sichuan 610031 China

**Keywords:** Auricular acupuncture, Insomnia, Systematic Review, GRADE

## Abstract

**Background:**

Primary insomnia is a common health issue in the modern world. We conducted a systematic review of the auricular therapy, aiming to evaluate whether there are advantages of auricular acupuncture with seed or pellet attachments for the treatment of primary insomnia.

**Methods:**

A search of relevant literatures was performed on major medical databases, including Medline, Embase, CENTRAL, CBM, CNKI, VIP, Wanfang Data and so on. Risk of bias evaluation, meta-analysis, sensitivity analysis and evidence rating of all extracted information were conducted also.

**Results:**

A total of 1381 records were identified, with 15 studies deemed eligible for the present review. Meta-analyses were conducted in two comparisons separately: participants received auricular acupuncture were more likely to make an improvement in clinical effective rate (RR = 1.40, 95% CI 1.07 to 1.83), sleep duration (MD = 56.46, 95% CI 45.61 to 67.31), sleep efficiency(MD = 12.86, 95% CI 9.67 to 16.06), global score on PSQI (MD = -3.41, 95% CI -3.93 to -2.89), number of awakenings( MD = -3.27, 95% CI -6.30 to -0.25) and sleep onset latency(MD = -10.35, 95% CI -14.37 to -6.33) when compared to sham auricular acupuncture or placebo; while in auricular acupuncture VS medications comparison, a better effective rate (RR = 1.24, 95% CI 1.15 to 1.34), better sleep efficiency(MD = 21.44, 95% CI 16.30 to 26.58), lower PSQI score (MD = -3.62, 95% CI -4.59 to -2.65) and less adverse effect (RR = 0.11, 95% CI 0.04 to 0.26) can be seen also in auricular acupuncture group. Although these results suggested benefits of auricular acupuncture, the overall quality of evidence rated by the GRADE system was low.

**Conclusion:**

Statistical analyses of the outcomes revealed a positive effect of auricular acupuncture for primary insomnia. Nonetheless, considering the poor methodological quality, insufficient sample size and possible publication bias, current evidence is not yet adequate to provide a strong support for the use of auricular acupuncture in the treatment of primary insomnia. More strictly designed clinical studies will be needed to obtain a more explicit conclusion.

**Electronic supplementary material:**

The online version of this article (doi:10.1186/s12906-015-0606-7) contains supplementary material, which is available to authorized users.

## Background

Primary insomnia is a common health issue caused by multiple environmental and psychological factors. Patients suffering from insomnia may have difficulty initiating or maintaining sleep, or experience nonrestorative sleep together with functional impairment during the daytime without a clear medical or psychiatric cause [1,2]. Approximately 10-20% of the population worldwide is suffering from poor-quality sleep, among which 50% involve a chronic course over a month [3]. Specifically, sleep disorder persisting one month is an acute type, between one to three months is a sub-chronic type, beyond three months could be defined as persistent insomnia [4]. The incidence of insomnia increases with age and is more likely to affect women than men [5,6]. In addition, it is frequently co-morbid with growing risk of headache [7], anxiety and depression [8,9], hypertension [10,11], cardio-cerebrovascular diseases [12,13], and even suicidal thoughts or behaviours [14,15].

Current understanding of the mechanism of insomnia typically involves arousal of the central and autonomic nervous systems [16]. For example, sympathetic hyperarousal has been detected in insomniacs, as evidenced by increased heart rate, heart rate variability, metabolic activation, and activation of the sympathetic nervous system during sleep [17-19]. Neuroimaging studies have also revealed changes in brain activity associated with the frontal cortex, hippocampus and anterior cingulate cortex [20,21].

There has been an adequate number of clinical studies supporting the use of cognitive behavioural therapy (CBT) and hypnotics for insomnia [4,22]. However, not all patients respond well to these treatments: experiencing adverse effects from or dependency on hypnotic would all potentially diminish the impact of treatments. Besides, information regarding the long-term efficiency of hypnotic drugs is currently unavailable [23]. Although CBT has been better accepted by insomnia patients, barriers such as cost of time and limited number of trained practitioners still exist in the implementation of CBT [4,24]. Consequently, complementary and alternative medicine (CAM) has become an option that insomnia sufferers often turned to [25,26].

In ancient China, the medical classic Yellow Emperor’s Canon of Medicine first described the complicated link between ears and internal organs, and indicated that any physiological or pathological changes inside the body may reflect on the auricles. In the 1950s, Dr. Paul Nogier of France developed and refined the modern theory of auricular acupuncture, which was based on the concept of an inverted fetus map on the external ear [27], suggesting a reflex system useful for the diagnosis and treatment of diseases [28]. Clinically pellet attachments are utilized on a large scale to provide perfect stimuli to the ears without piercing the skin. Pellets come in different materials, usually in magnetic pearls and plant seeds.

To date, many studies on auricular acupuncture for insomnia have been published worldwide. However, inclusion of low quality of evidence and inconsistency of outcomes of the studies present challenges in drawing a firm conclusion on the benefit of this therapy [29-31]. It is therefore worthwhile to conduct a systematic review with a more rigorous inclusion criteria and evidence rating system, aiming to evaluate the advantages of auricular acupuncture with seed or pellet attachments in the treatment of primary insomnia in a more explicit manner.

## Methods

### Database

The following databases were searched: Medline (1946 to November 2013), Embase (1946 to November 2013), PsycINFO(1950 to November 2013), Cochrane Central Register of Controlled Trials (CENTRAL, November 2013), Cochrane Methodology Register (CMR, 3rd Quarter 2013), Cochrane Database of Systematic Reviews (Cochrane DSR, 2005 to November 2013), ACP Journal Club (1991 to November 2013), Database of Abstract of Reviews of Effects (DARE, 3rd Quarter 2013), ProQuest Dissertations and Theses (PQDT, 1997 to 2013), International Clinical Trial Registry Platform (ICTRP), Centre for Clinical Trials Clinical Trials Registry (CCTCTR), and Chinese Biomedical Database (CBM, 1978 to September 2013), China National Knowledge Internet (CNKI, 1915 to September 2013), VIP Database (1989 to September 2013), Wanfang Data and Wanfang Dissertation Database (1989 to June 2013). A monthly e-mail alert was also set up at the National Center for Biotechnology Information (NCBI) from the U.S. National Library of Medicine (NLM), to obtain updates of new publications.

### Search strategy

1. auricular acupuncture, ear acupuncture or acupuncture ear in full text;
2. auricular therapy, auriculotherapy, auricular needle, auricular acupressure, otopoint$, otoneedle, auriculoacupuncture$ or otopuncture$ in title and abstract;
3. 1 or 2
4. sleep or insomnia in full text;
5. sleep$, insomnia$ ,wakeful$ , somnambul$ or somnipathy$ in title and abstract;
6. 4 or 5
7. 3 and 6

**(Additional file 1-Search Strategy)** was supplemented by hand searching the reference lists from identified reviews and original articles in the full text.

### Inclusion criteria

1. Design: randomized controlled trials only.
2. Participants: age between 18 ~ 80 with dissatisfaction about sleep quality of longer than one month’s duration [32-34].
3. Intervention: plant seeds, metal pellets, or magnets attached on the auricles was suppose to be the single intervention in treatment groups; while sham auricular acupuncture, placebo, no treatment, or drug therapy could be adopted in the control arms.
4. Primary outcome
  Effective rate, defined as the proportion of participants who had improved obviously like total sleep time prolonged, sleep efficiency or sleep quality improved, or symptoms relieved, etc. This is a subjective assessment of overall effectiveness and ought to be reported by participants themselves.
5. Secondary outcomes
  Sleep parameters, measured by either subjective or objective approaches, e.g., sleep diary, actigraphy, electroencephalogram (EEG) or polysomnogram (PSG);
  Sleep efficiency, the ratio of total sleep time to time in bed, could be derived from either subjective or objective ways as well;
  Scales or index for sleep quality evaluation, e.g., the Pittsburgh Sleep Quality Index(PSQI), Insomnia Severity Index (ISI), Athens Insomnia Scale (ASI) et al.;
  Adverse effect, reported in the articles or measured by validated scales, e.g., Health Survey Questionnaire, Treatment Emergent Symptom Scale (TESS) et al.
6. Language restriction: English and Chinese records only.

### Exclusion criteria

Studies that compared different auricular point prescriptions or stimulations in parallel groups were excluded. Studies without mention about randomization were excluded. Redundant publications were excluded.

### Data collection and analysis

Two review authors were independently responsible for article screening and data extraction; the third one verified all information. Disagreements were resolved by consensus. The data extraction form was designed previously meeting the Cochrane standard [35]. The authors attempted to contact the original authors of all included articles for further information by email or telephone.

The formulae from *Cochrane Handbook for Systematic Reviews of Interventions (Handbook)* Chapter 7.7.3.8 were adopted to combine data from two subgroups into one, if more than two parallel groups appeared.

Assessment of risk bias was in accordance with *Handbook*, in which a domain-based evaluation method was highly recommended. We designated the following seven domains for each study: random sequence generation, allocation concealment [36], blinding personnel and participants, blinding assessor, incomplete data outcome, selective reporting, and other bias.

### Summary statistics

We chose risk radio (RR) for dichotomous data and mean difference (MD) for continuous data, with 95% confidence intervals (CI) in the meta-analysis. For heterogeneity evaluation, we employed the Q (χ^2^) test and I^2^ statistics. Fixed effect model was used to calculate summary statistics in the absence of substantial heterogeneity (I^2^ < 40%). Conversely, random effect model was chosen when I^2^ > 40%. All meta-analyses results were presented according to different control methods separately. If possible, we planned to conduct subgroup analyses or sensitivity analyses for different diagnostic criteria, age groups, course of disease to assess robustness of all the outcomes.

We planned to generate funnel plots for publication bias assessing when more than ten studies reported the same outcome and use the GRADE system for evidence rating [37]. Quality of evidence for each outcome was ranked at one of four levels, namely high, moderate, low or very low level based on five downgrading factors [38-42] and three upgrading factors [43].

Review Manager 5.2.5 was utilized for statistical analysis, GRADEprofiler 3.6 for evidence profile making. We presented this review under the guidance of Preferred Reporting Items for Systematic reviews and Meta-Analyses (PRISMA) [44,45].

## Results

**Figure 1:**
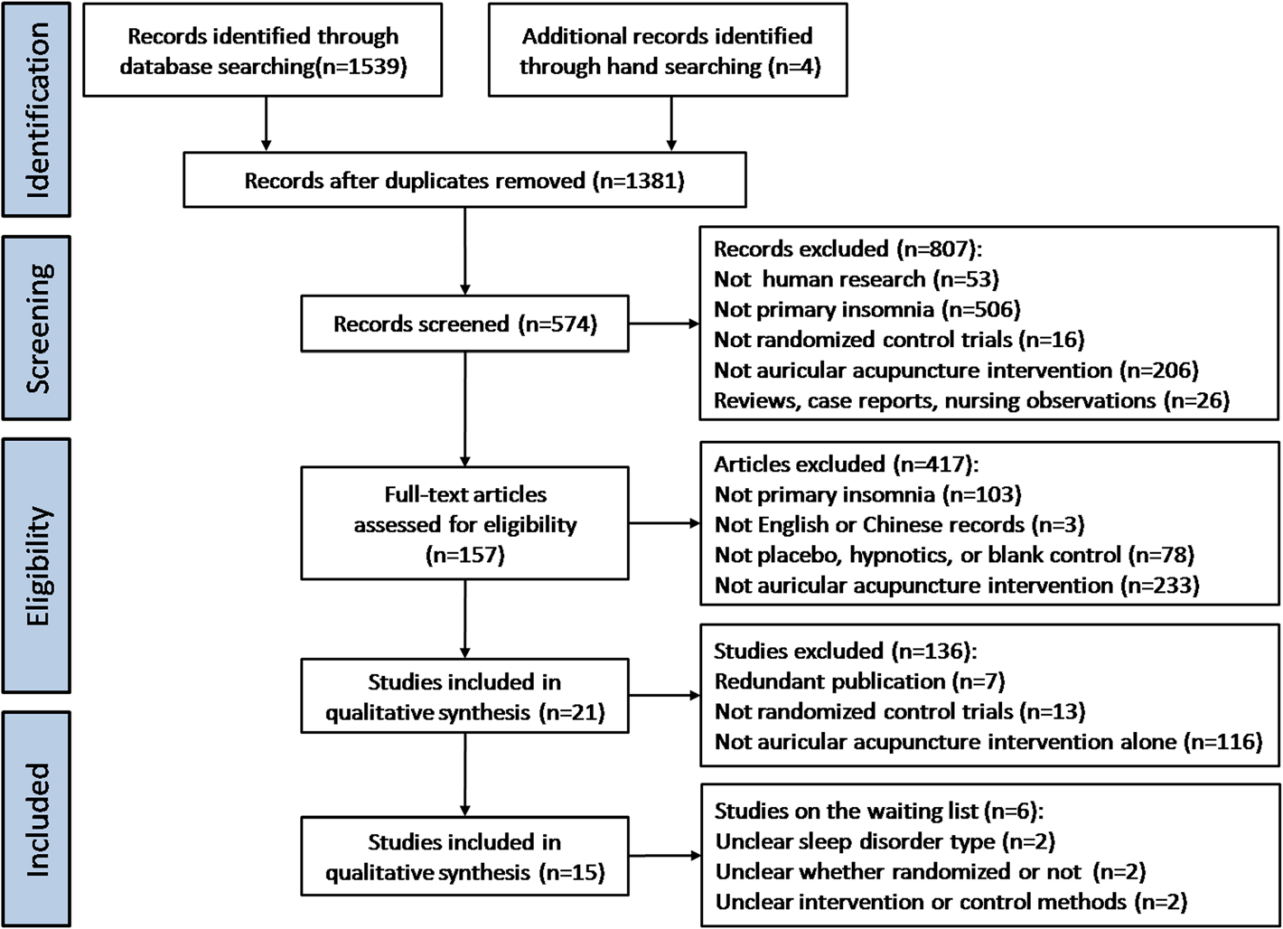
**Flow of information through the different phases of systematic review.**

Finally, 15 out of 1381 records were identified. They were performed in mainland China [46-56], Hong Kong [57], Taiwan [58,59], and the US [60], from 1993 to 2013 (Figure 1).

All together 1429 participants were included, aged from18 to 78, 510 male, with insomnia duration from one month to more than ten years. Of these participants, 737 received auricular acupuncture and 664 received control method. Sample sizes ranged from 21 to 300, yet calculation methods weren’t revealed in any of these articles.

Diagnosis of insomnia was made using the following criteria: International Classification of Disease, 10th Version(ICD-10) [60], Chinese Classification of Mental Disorders(CCMD) [46-49,51,52,55,59], Clinical Research Guidelines of New Chinese Herbal Medicine [55,56,59], Chinese Medicine Clinical Syndrome Diagnostic Criteria [54,59], sleep efficiency [55,57-60], Pittsburgh Sleep Quality Index (PSQI) [47,48,50-52,58] and self-report of enduring poor sleep [53].

In the treatment groups, seven studies [46-48,51-53,59] picked Semen Vaccariae for auricular attachment, six [49,50,54,56,58,60] chose magnetic pearls, one [57] study used seeds in one group and magnetic pearls in a second group, and only one study [55] did not report the type of material for attachment. While in the control groups, two studies [48,52] chose sham auris-points method, two used [50,59] pseudo plasters, three studies [57,58,60] treated participants with no pressure or additional force on the pellets or seeds; four utilized [49,51,55,56] estazolam, and four adopted [46,47,53,54] diazepam control. Eight studies [46-48,51,54,57,58,60] took a standard points protocol and the rest seven took individual prescriptions according to syndrome differentiation instead.

Six studies [46,47,49,55,56,59] assessed effects with Guiding Principle of Clinical Study on New Drug of Traditional Chinese Medicine, one study [54] adopted Criteria of Diagnosis and Therapeutic Effect of Interna1 Diseases and Syndromes in Traditional Chinese medicine, six studies [47,48,50-52,59] took the change value of sleep quality assessment scale, and only one [53] took self-made standard. Four studies [49,53,55,56] reported adverse events and two [57,60] reported follow up duration.

### Risk of bias

Of the fifteen included studies, six described the details of random sequence generation by computer software [50,52,60] or random number table [48,56,58], and merely one [60] conducted proper allocation concealment. Seven studies [48,50,52,57-60] used sham auricular acupuncture or placebo control to blind the participants. Three [48,57,60] out of fifteen studies reported drop-outs and two [57,60] with reasons, and one [48] carried out the intention-to-treat analysis. In addition, six studies [46,47,53,54,58,59] declared no drop-outs in their program. In selective reporting, two studies [51,59] failed to report all parameters from PSQI scale, while three studies [50,54,57] lacked between-group baseline comparison (Table 1).

**Table 1:** **Characteristics of Included studies**

**Study**	Sample Size*		**Randomized Method**	**Treatment**	**Control**	**Course**	**Outcome Measurements**	**Follow-Up**	**Safety**	**Assessment**
	**(Male/Female)**									of Bias**
Chen 2012 [46]	16/20	14/22	NR	SV	Diazepam	30 Days	Effective rate	NR	NR	?,?,-,?,+,?,?
Chyi Lo 2013 [58]	0/14	0/13	Random Number Table	MP	Sham AA	>21 Days	PSG, PSQI	NR	NR	+,?,+,-,+,+,-
Hisghman 2006 [60]	1/10	1/9	Computer Software	MP	Stainless Steel Pellet	17 Days	PSQI Sleep Diary, ISI, MOS SF-12, TEQ	12 Days	NR	+,+,+,?,+,+,-
Hu 2010 [47]	22/10	18/13	NR	SV	Diazepam	28 Days	Effective rate, PQSI, AIS	NR	NR	+,-,-,-,+,-,-
Jiang 2010 [48]	14/49	10/52	Random Number Table	SV	Sham AA	20-25 Days	PSQI	NR	NR	+,-,+,-,+,+,-
Jin 2012 [49]	13/27	15/25	NR	MP	Estazolam	7-14 Days	Effective rate, TESS	NR	Reported	+,-,-,?,?,?,?
Lin 2007 [59]	16/14	17/13	NR	SV	Sham AA	51 Days	Effective rate, EEG, PQSI	NR	NR	?,?,+,?,+,-,-
Liu 2008 [50]	26/74	37/63	Computer Software	MP	Sham AA	30 Days	PSQI	NR	NR	+,?,+,?,-,-,-
Luo 2010 [51]	11/10	9/12	NR	SV	Estazolam	30 Days	Effective rate, PQSI	NR	NR	?,?,-,?,-,-, -
Pi 2012 [52]	71/79	75/75	Computer Software	SV	Sham AA	8 Weeks	PSQI	NR	NR	+,-,+,-,-,-,-
Suen 2002 [57]	10/110***		NR	MP/SV	Junci Medulla Attachment	21 Days	Sleep Diary, Actigraphy	0.5-1 Year	NR	?,?,+,+,+,-,-
Wang 1993 [53]	10/20	12/18	NR	SV	Diazepam	20 Days	Effective rate, SCL-90	NR	Reported	?,?,-,?,+,-,-
Wang 2002 [54]	17/13	14/16	NR	MP	Diazepam	3-10 Days	Effective rate	NR	NR	?,?,-,?,+,-,-
Wei 2010 [55]	22/14	21/15	NR	NR	Estazolam	28 Days	Effective rate, Sleep Efficiency	NR	Reported	?,?,-,?,?,?,-
Zhang 2008 [56]	10/58	8/50	Random Number Table	MP	Estazolam	18 Days	Effective rate	NR	Reported	?,?,-,?,?,?,-

*Abbreviations: NR* Not Reported, *SV* Semen Vaccariae Attachment, *MP* Magnetic Pearls Attachment, *AA* Auricular Acupuncture,* PGS *Polysomnography, *PSQI* Pittsburgh Sleep Quality Index, *MOS SF-12* Medical Outcomes Study Short Form-12, *TEQ *Treatment Expectancy Questionnaire, *AIS* Athens Insomnia Scale, *TESS* Treatment Emergent Symptom Scale, *EEG* electroencephalogram, *SCL-90* Symptom CheckList-90. *Sample Size: sample size of treatment groups are on the left column, control groups on the right. **Assessment of Bias: the seven domains were random sequence generation, allocation concealment, blinding personnel and participants, blinding assessor, incomplete data outcome, selective reporting, and other forms of bias from left to right. +: low risk, - : high risk, ?: *** In study Suen 2002, the male to female ratio was not reported after grouping.

### Effect estimates

According to various control methods tested in the trials, we conducted meta-analyses in two comparisons respectively: *auricular acupuncture VS sham or placebo method*, and *auricular acupuncture VS medications*. Since outcome measurements varied in studies, effect estimates on outcome measurements were different, and then not all fifteen studies appeared in each meta-analyses.

### Effective rate

This is a dichotomous outcome and a subjective assessment of overall effectiveness, defined as the proportion of participants who had improved obviously in sleep quality.

### Auricular acupuncture VS sham or placebo method

**Figure 2:**
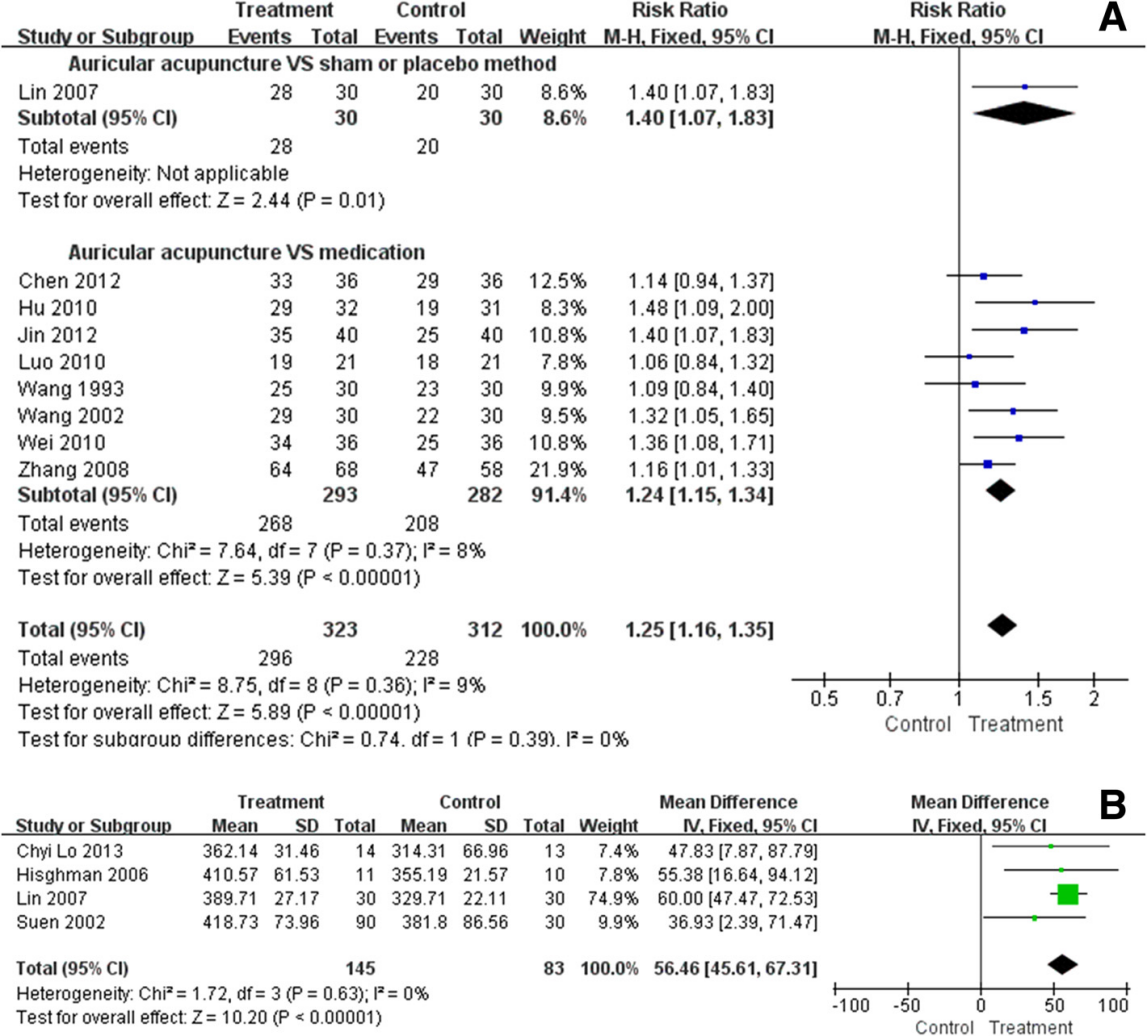
**The pooled outcomes of effective rate and total sleep time revealed a better therapeutic effect of auricular acupuncture, statistically. A**. Forest plot of effective rate; Proportion of patients had positive response to the intervention. **B**. Forest plot of total sleep time; Time in bed minus total awake time, better indicated by higher values, longer than six hours supposed to be normal.

One study [59] reported that the auricular acupuncture group had better effective rate compared to the sham acupuncture (RR = 1.40, 95% CI 1.07 to 1.83, P = 0.01). (Figure 2A) But this study did not have sufficient power to prove the superiority of auricular acupuncture over sham or placebo method because the sample size was not enough.

### Auricular acupuncture VS medications

Eight studies [46,47,49,51,53-56] reported the effective rate after intervention. The pooled results showed that the auricular acupuncture group was more likely to improve the sleep effective rate when compared to the medication (RR = 1.24, 95% CI 1.15 to 1.34). (Figure 2A) The mild heterogeneity (χ^2^ = 7.64, P = 0.37, I^2^ = 8%) might be because of different evaluation criteria for the effective rate. Therefore, we carried out a sensitivity analysis without those referring to other criteria to confirm whether the heterogeneity was due to different criteria, and remaining five studies [46,47,49,55,56] all used Chinese Classification of Mental Disorders(CCMD) [61] for effective rate evaluation. The pooled result of these five still confirmed an advantage of auricular acupuncture over medications (RR = 1.27, 95% CI 1.16 to 1. 40), indicating the outcome of original meta-analysis was stable and different criteria had few effects on it.

### Total sleep time *(minutes)*

Total sleep time is time in bed minus sleep onset latency and minus waking time after sleep onset. This is continuous data, better indicated by higher values. Longer than six hours is defined as one of the treatment goals [62].

### Auricular acupuncture VS sham or placebo method

Four studies reported the outcome measured by self-reported sleep diary [60], wrist actigraphy [57], EEG [59] and PSG [58]. Statistics showed that auricular acupuncture intervention was more likely to prolong total sleep time compared to the control group (MD = 56.46, 95% CI 45.61 to 67.31), with no heterogeneity (χ^2^ = 1.72, P = 0.63, I^2^ = 0%). (Figure 2B) In all treatment groups of these four studies, the total sleep time extended longer than six hours after intervention, indicating benefit from auricular acupuncture was clinically relevant.

### Auricular acupuncture VS medications

No studies in this comparison reported this outcome.

### Sleep efficiency *(%)*

Sleep efficiency, the percent of time asleep while in bed, is also continuous data and higher values indicate better sleep efficiency. Higher than 80% to 85% is defined as normal [62].

### Auricular acupuncture VS sham or placebo method

**Figure 3:**
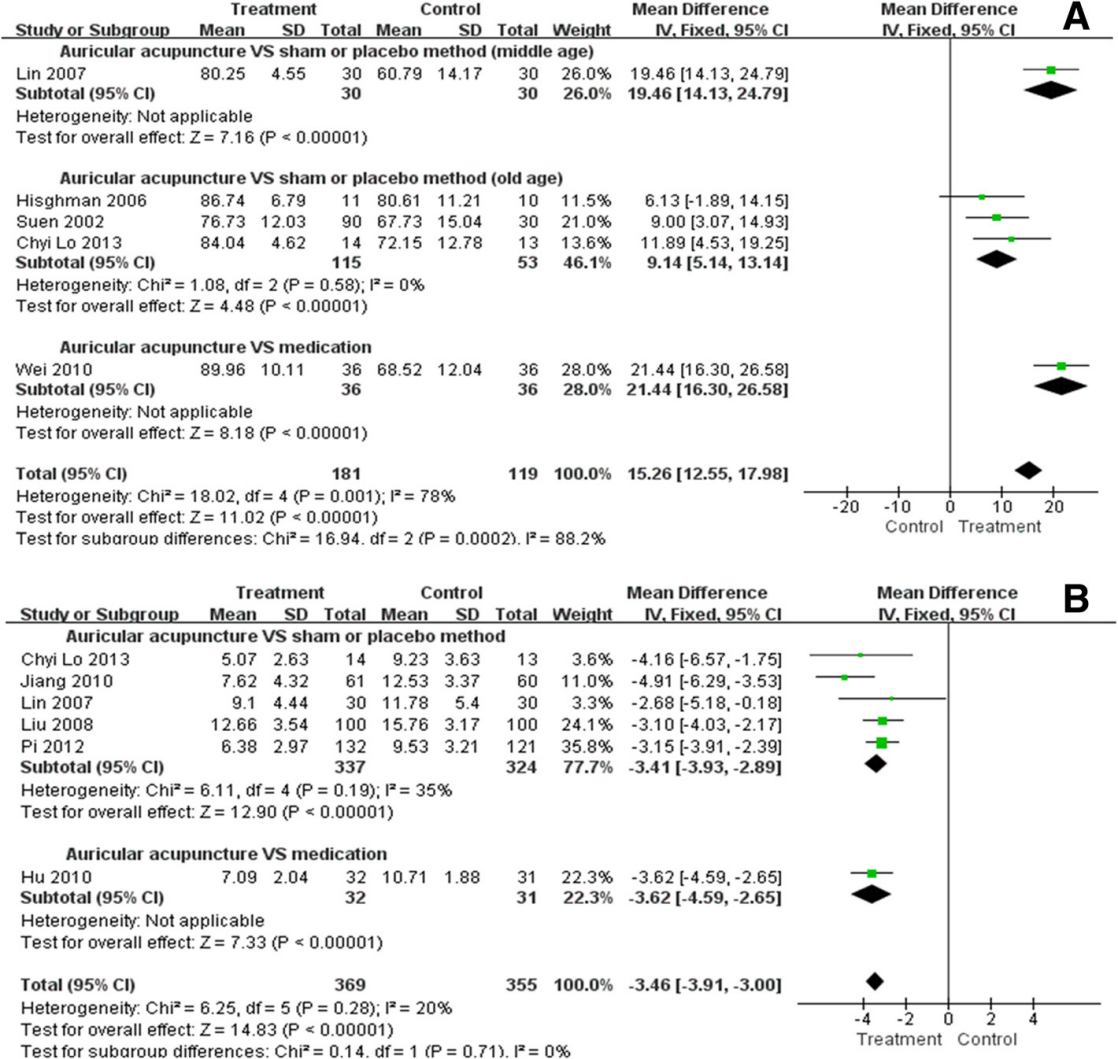
**The pooled outcomes of sleep efficiency and global score on PSQI revealed a better therapeutic effect of auricular acupuncture, statistically. A**. Forest plot of sleep efficiency; Percent of time asleep while in bed, higher values indicate better sleep efficiency, and higher than 80% to 85% is defined as normal. **B**. Forest plot of global score on PSQI; Range 0-21, clinically important effects were defined as a minimum decrease by at least 1.93 for a beneficial effect and an increase of at least 2.9 for a negative effect.

Due to the high heterogeneity of included studies MD = 12.86, 95% CI 9.67 to 16.06, χ2 = 10.30, P = 0.02, I2 = 71%, we introduced subgroups according to age. As we can see in the old age subgroup [57,58,60], sleep efficiency was comparatively higher in auricular acupuncture intervention (MD = 9.14, 95% CI 5.14 to 13.14), with no heterogeneity (χ^2^ = 1.08, P = 0.58, I^2^ = 0%). Also, there was a better improvement in the middle age group [59] (MD = 19.46, 95% CI 14.13 to 24.79) and the sleep efficiency was higher than 80% after treatment. These results indicated some benefit from auricular acupuncture, and it was more significant in the middle-aged than that in the old (Figure 3A).

### Auricular acupuncture VS medications

One study [55] reported that auricular acupuncture was superior to estazolam in sleep efficiency (MD = 21.44, 95% CI 16.30 to 26.58, P < 0.00001). (Figure 3A) This study however, was poor in methodological quality and failed to provide useful information about details, so this result needs further consideration.

### Global score on PSQI *(points)*

PSQI is a numeric rating scale range from 0 to 21. Its seven components yield one global score [63]. Higher score indicates worse sleep quality. Clinically important effects were defined as a decrease by at least 1.93 for a beneficial effect and an increase of at least 2.9 for a negative effect [64].

### Auricular acupuncture VS sham or placebo method

Five out of the fifteen studies [48,50,52,58,59] revealed the global score on PSQI, and pooled results were in favour of auricular acupuncture (MD = -3.41, 95% CI -3.93 to -2.89). The heterogeneity assessment result was also acceptable (χ^2^ = 6.11, P = 0.19, I^2^ = 35%). (Figure 3B) More importantly, the global score before and after intervention from all these treatment groups dropped more than 1.93, according to reported data, indicating the benefit of auricular acupuncture in sleep quality improvement was clinically relevant.

### Auricular acupuncture VS medications

Only one study [47] was eligible for the meta-analysis and revealed lower PSQI score after auricular therapy compared to diazepam (MD = -3.62, 95% CI -4.59 to -2.65, P < 0.00001). (Figure 3B) This study was also poor in quality and inadequate to support the outcome.

### Mean number of awakenings *(No.)*

Average number of mid-sleep awakenings is continuous data. The more frequent awakenings occurred, the poorer sleep could be.

### Auricular acupuncture VS sham or placebo method

**Figure 4:**
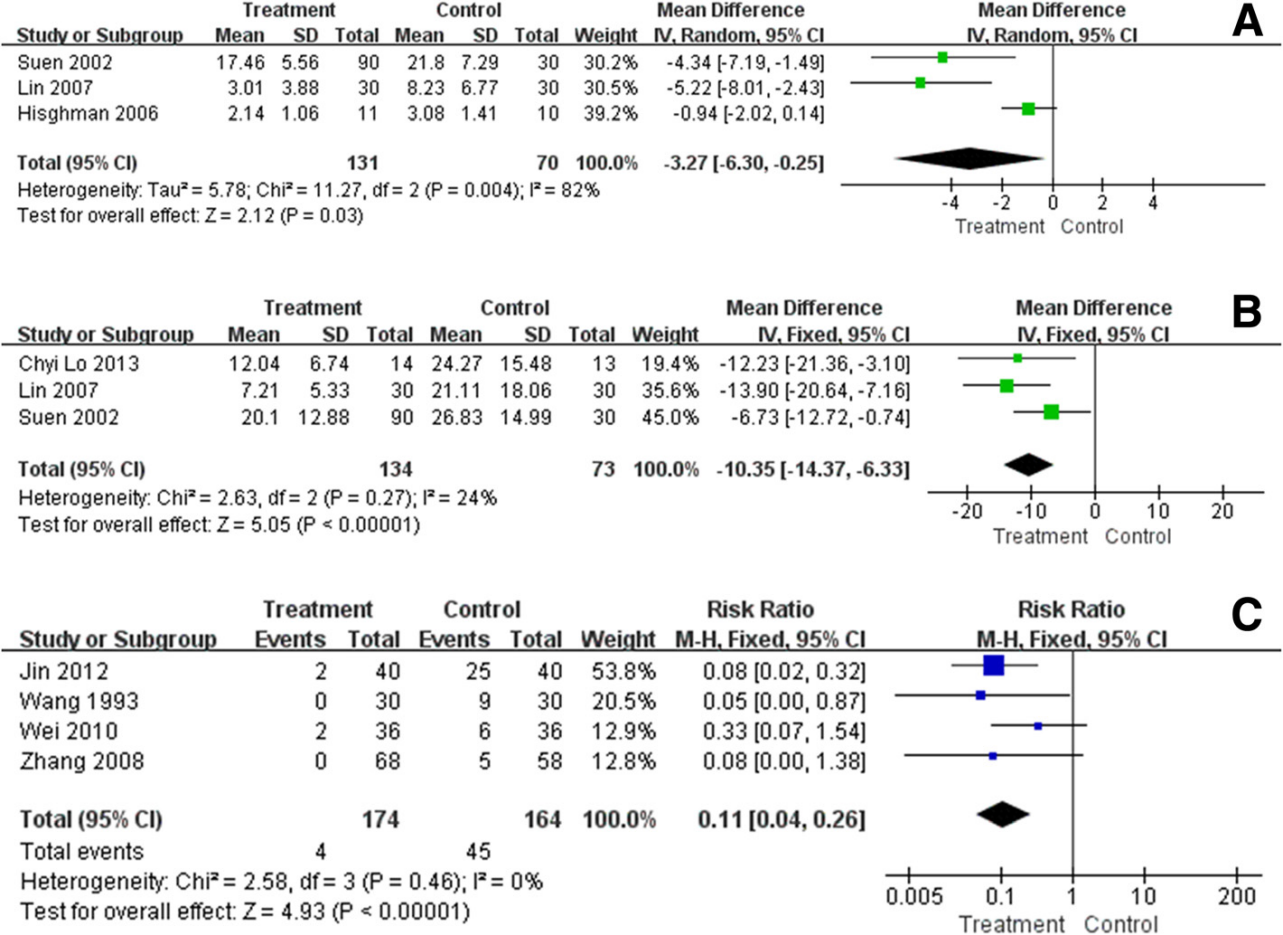
**The pooled outcomes of number of awakenings, sleep onset latency and adverse effects also revealed a better therapeutic effect of auricular acupuncture, statistically. A**. Forest plot of number of awakenings; Mean number of mid-sleep awakenings, the more frequently awakenings occurred, the poorer the sleep. **B**. Forest plot of sleep onset latency; Time in bed until falling asleep, less than 30 minutes is normal. **C**. Forest plot of adverse effects.

Three studies reported the number of awakenings as assessed by sleep diary [60], EEG [59] or wrist actigraphiy [57]. Since the substantial heterogeneity among studies (χ^2^ = 11.27, P = 0.004, I^2^ = 82%) cannot be resolved by subgroup analyses, pooled results were calculated with random model (MD = -3.27, 95% CI -6.30 to -0.25). (Figure 4A) Decreased frequency of awakenings reflected a better sleep quality after auricular acupuncture. The large heterogeneity might be caused by the difference in outcome measures used.

### Auricular acupuncture VS medications

No studies in this comparison reported this outcome.

### Sleep onset latency *(minutes)*

Sleep onset latency, the time in bed until falling asleep, is continuous data. Less than 30 minutes is defined as normal [62].

### Auricular acupuncture VS sham or placebo method

PSG [58], EEG [59], and wrist actigraphy [57] were adopted in three studies respectively and the pooled results showed sleep onset latency was shortened in the auricular acupuncture group compared to the control group (MD = -10.35, 95% CI -14.37 to -6.33). However, the difference of just ten minutes was not clinically relevant. The slight heterogeneity might be explained by the different age of participants included in studies (χ^2^ = 2.63, P < 0.00001, I^2^ = 24%) (Figure 4B).

### Auricular acupuncture VS medications

No studies in this comparison reported this outcome.

### Adverse effect

#### Auricular acupuncture VS sham or placebo method

No studies in this comparison reported this outcome.

### Auricular acupuncture VS medications

Four studies, taking estazolam or diazepam as the control, reported adverse events during intervention. Wei [55] reported two cases of auricle pain in treatment group, while the control group had two cases of headache, three of dizziness, one of thirst, and two of fatigue. Five cases of symptoms of a hangover, four of weakness, and one of poor coordination were reported in the control group from Wang’s study [53]. In Zhang’s study [56], five cases in the control group had daytime sleepiness. Jin [49] reported two cases using magnetic pearls experienced redness at the attached points and withdrew after treatment, as well as twenty-five cases experiencing drug dependence in the control group. In meta-analysis, RR was 0.11, 95% CI ranged from 0.04 to 0.26, and heterogeneity assessment turned out to be χ^2^ = 2.58, P < 0.00001, I^2^ = 0%, signalling a reliable outcome in favour of the intervention group. Results demonstrated that auricular acupuncture caused less frequent and less severe adverse events compared with conventional hypnotics (Figure 4C).

### Publication bias

As less than ten studies were included in a single outcome, the funnel plot would have no valuable reference.

### Evidence rating

Evidence rating would provide a transparent, concise illustration of decision making process in form of the GRADE evidence profile. Ten outcomes from meta-analyses were presented in two tables (Tables 2 and 3).

**Table 2:** **Grade evidence profile of auricular acupuncture vs sham or placebo method comparison**

**No. of studies**	**Design**	**Quality assessment**					**No. of patients**			**Effect**	**Quality**	**Importance**
		**Risk of bias**	**Inconsistency**	**Indirectness**	**Imprecision**	**Other**	**Treatment**	**Control**	**Relative (95% CI)**	**Absolute**		
**Effective Rate (Measured with: CCDM-3 )**												
1	RCT	no serious limitation	serious^2^	no serious indirectness	serious^4^	none^6^	28/30 (93.3%)	20/30 (66.7%)	RR 1.40 (1.07 to 1.83)	267/1000 (47 ~ 553)	LOW	IMPORTANT
**Total Sleep Duration** *minutes* **(Measured with: EEG, PSQI Sleep Diary, or wrist actigraphy; Better indicated by higher values)**												
4	RCT	serious^1^	no serious inconsistency	no serious indirectness	serious^5^	none^6^	145	83	—	MD 56.46 (45.61 ~ 67.31)	LOW	CRITICAL
**Sleep Efficiency** *%* **(Measured with: International Standard Sleep Efficiency; Better indicated by higher values)**												
4	RCT	serious^1^	serious^3^	no serious indirectness	serious^5^	none^6^	145	183	—	MD 12.86 (9.67 ~ 16.06)	VERY LOW	CRITICAL
**Global Score On Psqi** *points* **(Measured with: PSQI; range of scores: 0-21; Better indicated by lower values)**												
5	RCT	serious^1^	no serious inconsistency	no serious indirectness	no serious imprecision	none^6^	337	325	—	MD -3.41 (-3.93 ~ -2.89)	MODERATE	IMPORTANT
**Mean Number Of Awakennings** *No.* **(Measured with: EEG, PSQI Sleep Diary, or wrist actigraphy; Better indicated by lower values)**												
3	RCT	serious^1^	serious^3^	no serious indirectness	serious^5^	none^6^	131	70	—	MD -3.27 (-6.30 ~ 0.25)	VERY LOW	IMPORTANT
**Sleep Onset Latency** *minutes* **(Measured with: EEG, PSQI Sleep Diary, or wrist actigraphy; Better indicated by lower values)**												
3	RCT	serious^1^	no serious inconsistency	no serious indirectness	serious^5^	none^6^	134	73	—	MD -10.35 (-14.37 ~ -6.33)	LOW	IMPORTANT

1. Methodological quality of included studies was not high.

2. With only one study involved, there were no heterogeneity assessment results for evaluation.

3. The pooled outcome revealed high heterogeneity.

4. The pooled (cumulative) sample size was lower than the optimal information size (OIS) and/or the total enrollment was less than 400 (continuous data).

5. The pooled (cumulative) sample size was lower than the optimal information size (OIS) and/or the total enrollment was less than 300 (dichotomous data).

6. The number of included studies is not enough to estimate publication bias temporarily.

*Abbreviations:*

95% CI: 95% Confidence Interval.

CCMD-3: Chinese Classification of Mental Disorders, 3^rd^ version;

MD: Mean Difference

EEG: Electroencephalogram.

PSQI: Pittsburgh Sleep Quality Index.

RR: Risk Radio.

**Table 3:** **Grade evidence profile of auricular acupuncture vs medications comparison**

**No. of studies**	**Design**	**Quality assessment**					**No. of patients**			**Effect**	**Quality**	**Importance**
		**Risk of bias**	**Inconsistency**	**Indirectness**	**Imprecision**	**Other**	**Treatment**	**Control**	**Relative (95% CI)**	**Absolute**		
**Effective Rate (Measured with: CCDM-3,PSQI et al.)**												
8	RCT	very serious^1^	no serious inconsistency	no serious indirectness	no serious imprecision	none^4^	268/293 (91.5%)	208/282 (73.8%)	RR 1.24 (1.15 ~ 1.35)	177/1000 (111 ~ 258)	LOW	CRITICAL
								61.29%		147/1000 (92 ~ 215)		
								85.71%		206/1000 (129 ~ 300)		
**Sleep Efficiency** *%* **(Measured with: International Standard Sleep Efficiency; Better indicated by higher values)**												
1	RCT	serious^1^	serious^2^	no serious indirectness	serious ^3^	none^4^	36	36	—	MD 21.44 (16.30 ~ 26.58)	VERY LOW	IMPORTANT
**Global Score On PAQI** *points* **(Measured with: PSQI; range of scores: 0-21; Better indicated by lower values)**												
1	RCT	serious^1^	serious^2^	no serious indirectness	serious ^3^	none^4^	32	31	—	MD -3.62 (-4.59 ~ -2.65)	VERY LOW	CRITICAL
**Adverse Effect (Assessed with: SCL-90, TESS et al.)**												
4	RCT	very serious^1^	no serious inconsistency	no serious indirectness	no serious imprecision	none^4^	4/174 (2.3%)	45/164 (27.4%)	RR 0.11 (0.04 ~ 0.26)	244/1000 (203 ~ 263)	LOW	IMPORTANT
								8.62%		77/1000 (64 ~ 83)		
								62.50%		556/1000 (463 ~ 600)		

1. Methodological quality of included studies was not high.

2. With only one study involved, there were no heterogeneity assessment results for evaluation.

3. The pooled (cumulative) sample size was lower than the optimal information size (OIS) and/or the total enrollment was less than 400 (continuous data).

4. The number of included studies is not enough to estimate publication bias temporarily.

*Abbreviations:*

95% CI: 95% Confidence Interval.

CCMD-3: Chinese Classification of Mental Disorders, 3rd version.

MD: Mean Difference.

PSQI: Pittsburgh Sleep Quality Index.

SCL-90: Symptom Check List-90.

TESS: Treatment Emergent Symptom Scale.

RR: Risk Radio.

## Discussion

### Summary of main outcomes

We found auricular acupuncture showed benefit in all meta-analyses with significant differences between treatment groups and control groups. It improved the clinical effective rate, total sleep time, sleep efficiency, and lowered PSQI score, shortened sleep onset latency, and reduced number of awakenings when compared to sham auricular acupuncture or placebo. Also, it improved effective rate and sleep efficiency, lowered PSQI score, reduced the incidence of adverse effects when compared to conventional medications. But some of these statistical differences were not clinically relevant.

It is worth noting that, all included studies took either magnetic pearls or Semen Vaccariae for auricular pressing, the top two in clinical use. Some ear acupuncturists believe that it is not just choices of the modality of stimulation that matter but also the choice of material [65]. Magnetic pearls will place a magnetic field over biological tissues, when ions in the blood flow through magnetic lines, the electromagnetic interaction will happen, thus improving the circulation of blood. This idea was confirmed by previous studies that using magnetic pearls for insomnia [57,66]. For the purpose of this review, our primary concern is whether the therapeutic effect of auricular acupuncture stands out, instead of the material being use. So we didn’t plan subgroup analyses for magnetic pearls or Semen Vaccariae.

Although meta-analyses emphasised the positive aspect, the evidence rating was quite different. We evaluated ten outcomes through the GRADE system and presented results in two evidence profiles: in *auricular acupuncture VS sham or placebo method* comparison, one ranked moderate, three ranked low and two ranked very low; in *auricular acupuncture VS medications* comparison, two ranked low and two ranked very low. The most common reasons for downgrade lay in risk of bias and imprecision, for the majority of included studies were poorly designed or reported. They neglected crucial details for methodological evaluation [40], and their cumulative sample size failed to reach an appropriate level [41].

### Comparison with other reviews

Previous systematic reviews on auricular acupuncture for insomnia [29,67] all reported that evidence was inadequate to make a firm conclusion. One more recent review [68] utilizing auricular acupuncture as an adjuvant method concluded that body and auricular acupuncture combined was superior to body acupuncture alone. Moreover, systematic reviews [31,69-71] on a broader range taking all acupuncture modalities into account were conservative about the clinical relevance of auricular acupuncture. These inconsistencies were apparently owing to poor quality of methodology, weakness of data, high levels of heterogeneity and publication bias.

In comparison, our work narrowed the inclusion criteria to studies in which auricular acupuncture was the sole intervention. Then, we chose primary insomnia sufferers with at least one month of sleep problem as target population. Because secondary insomnia patients varied in physical conditions, symptoms and might result in substantial clinical heterogeneity in meta-analyses. Additionally, outcomes selected in the meta-analyses were of credible reliability and validity: five from the *auricular acupuncture VS sham or placebo method* comparison were all continuous variable, measured by PSG, EEG, actigraphic monitoring, PSQI, or Sleep Diary. These are internationally accepted indexes of sleep evaluation [72-74] and particularly the former three are objective sleep parameters; the adverse effects from the *auricular acupuncture VS medications* comparison have complemented the assessment from another point of view. Likewise, the GRADE system would help to evaluate each outcome not only statistically but also methodologically by revealing and detailing all defects, which will definitely facilitate a better understanding and improvement for further research.

### Implication of findings

With many countries reporting a high incidence of sleep disturbance in children and adolescents [75,76], insomnia appears to be a significant public health issue requiring accurate diagnosis and proper management. Our findings suggest auricular acupuncture is beneficial to primary insomnia sufferers in respect of sleep quality and quantity.

Modern studies on the mechanism of the interaction of seed or pellet attachment on the ear proved the underlying principles. Since the ear is richly innervated, the afferent projections from the auricular branch of the vagus nerve to the solitary tract nucleus formed the anatomical basis for the neuroregulation of auricular acupuncture [77]. On applying pressure, a nerve impulse could be received by both autonomic nervous system and central nervous system. At the same time, magnetic fields were added if magnetic pearls were attached. In traditional Chinese medicine theory this effect might promote the circulation of Qi and blood, therefore improving the physiological condition of the body.

The results of these studies explained the overall improvement of sleep parameters after intervention: longer total sleep time, higher sleep efficiency and clinical effective rate, lower PQSI score, shorter sleep onset latency, less awakening times and adverse events. However, methodological assessments indicated a lack of sufficient evidence to support a strong recommendation. More strictly designed clinical trials are required in order to facilitate an explicit conclusion on the benefits of auricular acupuncture for primary insomnia.

### Limitation

Several limitations in this review should be noted. First, only four of the included studies [53,57,58,60] referred standard ear maps. Currently, it is a major problem in research of auricular therapy both in western and eastern medical systems [27,78,79]. Diversity in point location may lead to different clinical outcomes, misunderstanding in medical research and academic communication, as well as ambiguous conclusions in a systematic review. Second, RCTs with sham auricular acupuncture, placebo or blank control were inadequate in quantity. As a compromise, we extended the inclusion criteria for trials taking medications as control and presented results according to separate control methods [80]. Third, we pooled data from Semen Vaccariae or magnetic pearls groups together in meta-analyses because studies with same material were limited. However, we believe it will be more rigorous to perform subgroup analyses or sensitivity analyses if data permitted. Additionally, we only conducted a search for Chinese and English articles so that inclusion bias was unavoidable.

## Conclusions

Auricular acupuncture has been extensively adopted in Asian countries. With minimal consumption of medical resources, auricular acupuncture could be integrated into routine care for insomnia without difficulty in a wide range of clinical settings. In this review, meta-analyses of all included studies revealed a positive effect of auricular acupuncture for primary insomnia, however the poor methodological quality, insufficient sample size and possible publication bias of these studies mean that the evidence is not yet adequate to provide a strong support for the therapy in primary insomnia management. Future experimental designs and publications need to be more specific in details [81], such as implementation, point selection and location, extension and time of stimulation. We hope this review will raise attention to this cost-effective, safe therapy and produce more scientific evidence to support its clinical application.

## Additional file

Additional file 1:
**Search Strategy for Medline, Embase and CBM.**
