# Supplementary material for: Auricular acupuncture with seed or pellet attachments for primary insomnia: a systematic review and meta-analysis
Source: BMC Complement Altern Med. 2015 Apr 2;15:103. doi: 10.1186/s12906-015-0606-7 (PMC4425871; doi:10.1186/s12906-015-0606-7)
Supplement: Additional file 1: — Search Strategy for Medline, Embase and CBM. [file 12906_2015_606_MOESM1_ESM.docx]

# Additional files

1. Search strategy for **Medline**
2. exp auricular acupuncture/ or exp ear acupuncture/ or acupuncture ear/
3. (auricular therapy or auriculotherapy or auricular needle or auricular acupressure or otopoint$ or otoneedle or auriculoacupuncture$ or otopuncture$).af
4. 1 or 2
5. exp sleep/or insomnia/
6. (sleep$ or insomnia$ or wakeful$ or somnambul$ or somnipathy$).af
7. 4 or 5
8. 3 and 6
9. Search strategy for **Embase**

#1. 'acupuncture'/exp

#2. otopoint:ab,ti

#3. otoneedle:ab,ti

#4. otopuncture:ab,ti

#5. auriculotherapy:ab,ti

#6. 'auricular needle':ab,ti

#7. 'auricular acupressure':ab,ti

#8. 'auricular acupuncture':ab,ti

#9. 'auricular therapy':ab,ti

#10. #1 OR #2 OR #3 OR #4 OR #5 OR #6 OR #7 OR #8 OR #9

#11. 'insomnia'/exp

#12. 'wakefullness'/exp

#13. somnipathy:ab,ti

#14. somnambul:ab,ti

#15. #11 OR #12 OR #13 OR #14

1. Search strategy for **CBM**

#1 缺省[智能]：耳穴-限定；随机对照试验；人类

#2 缺省[智能]：耳针-限定；随机对照试验；人类

#3 缺省[智能]：耳压-限定；随机对照试验；人类

#4 缺省[智能]：耳穴压丸-限定；随机对照试验；人类

#5 缺省[智能]：耳穴刺激-限定；随机对照试验；人类

#6 缺省[智能]：耳穴疗法-限定；随机对照试验；人类

#7 缺省[智能]：耳穴贴压-限定；随机对照试验；人类

#8 缺省[智能]：耳穴压豆-限定；随机对照试验；人类

#9 缺省[智能]：耳穴按摩-限定；随机对照试验；人类

#10 缺省[智能]：耳穴埋籽-限定；随机对照试验；人类

#11 （#1 OR #2 OR #3 OR #4 OR #5 OR #6 OR #7 OR #8 OR #9 OR #10）

#12 缺省[智能]：失眠-限定；随机对照试验；人类

#13 缺省[智能]：不寐-限定；随机对照试验；人类

#14 缺省[智能]：不得眠-限定；随机对照试验；人类

#15 缺省[智能]：不得卧-限定；随机对照试验；人类

#16 缺省[智能]：目不瞑-限定；随机对照试验；人类

#17 缺省[智能]：睡眠障碍-限定；随机对照试验；人类

#18 （#12 OR #13 OR #14 OR #15 OR #16OR #17）

#19 #11 AND #18
